# Event and Comment

**Published:** 1911-09-15

**Authors:** 


### DENTAL REGISTER.

**Vol. LXV. September 15, 1911. No. 9**

### EVENT AND COMMENT.

AMERICAN DENTISTRY ARRAIGNED.—The August Number of *Science and Discovery* contains an abstract with editorial comment of an article contributed to the *London Lancet* by Dr. William Hunter, Pathologist to London Charring Cross Hospital, which severely criticises American dentistry and the profession. In brief he maintains that American dentistry wholly disregards asepsis. He says we are not educated to understand the importance of asepsis, and that the most illustrious dentists are the worst sinners, because they have patients who want and are willing to pay for good displays of crown and bridge work. He enumerates a long list of diseases which owe their origin to, or are gravely complicated by oral sepsis, produced by badly done fillings, crowns and bridges, etc. He says it should be called "Septic Dentistry" rather than "Conservative Dentistry." He says that it is surgical malpractice to gold-cap either a healthy or a diseased tooth. He claims that infection is introduced into the system by teeth diseased from bad dental work which has not taken into account aseptic methods. He claims to have traced tonsilitis, gastric and intestinal infections, appendicitis, septicemia, nephritis, pernicious anemia and various nervous affections to bad crown and bridge work. He finally says American dentists are not educated properly, they do too much technical work and too little scientific work. "He goes about his work in the densest ignorance of infection and therapeutic." Altogether it is a most uncomplimentor arraignment.

We do not have access to the original article, and so cannot present a definite opinion in favor of or against the specific arraignment. We are inclined to say at once that Dr. Hunter would have serious difficulty to justify on a scientific and judicious basis the truth of the statements he is quoted as having made. They do not read like the statements of one who has investigated sufficiently to have reached scientific conclusions. The quotations read like grossly exaggerated or sensational statements and tends to excite our resentment.

We do not mean to say that there is no such thing as poor American Dentistry any more than we would maintain that there is no poor American surgery or theology. We do, however, most emphatically deny the sweeping statements that American Dentistry is "vicious both in principle and practice," and that "the members of the dental profession are uneducated other than in the mere handicraft of this art." There are all grades of dental colleges, the same as in medicine, law and other liberal professions; but the lowest grade dental school in this country gives over one-third of the time allotted to its course to the study of such medical science subjects as chemistry, anatomy, bacteriology, physiology, histology and pathology, and the subjects are all taught and studied as carefully as these subjects are in medical schools of similar grade. What is more, these branches are studied not only as pure sciences, but also as technically applied sciences. Dr. Hunter evidently knows little about the curriculum of the American Dental College or he would not make such a statement as that attributed to him, namely, "that the American Dentist is no student, and in no sense is he a man of science." We are inclined to believe Dr. Hunter has limited his observations to the loudly advertized so-called American Dentists of England, nine-tenths of whom never saw the inside of an American Dental College. They are impostors of the worst type and are undoubtedly capable of committing the criminal operations the Doctor in his ignorance attributed to the highest class American Dentist. All those quacks are bridge-workers and make great displays of gold teeth for the money they get from them, a thing no honest and properly educated American dentist would do or approve.

There are also, we are sorry to admit, bunglers in the dental profession who have been properly educated and should know better; but who from laziness or avarice stultify themselves and discredit their profession by inferior service to their patients. We do not believe, however, that this characterizes dentistry more than other callings. There are dentists who may be doing their work in such a manner as to justify Dr. Hunter's censure, but they are outside the pale of the great mass of honorable and ethical practitioners who are doing relatively as much to promote the happiness and welfare of humanity as any other body of men in the world. We know hundreds of men who are practicing dentistry as honestly and consistently and intelligently as any clergyman follows his calling, and we are not sure but they are saving as many souls as well as bodies among their patients.

The charges made by Dr. Hunter every intelligent dentist will rightfully scorn, for he knows that the very first matter taught the student of dentistry in these days is the importance of cleanliness. In fact the student is forced to recognize the grave consequences that may follow the lack of thorough cleanliness in treating teeth, because in the large majority of cases handled carelessly, infection will cause so much disturbance, in a few hours, after an operation, that the student is kept continually on his guard and realizes early that he must handle all cases so as to eliminate the least possible chance for infection. It is true there are cases where infectious results may not be sufficiently obvious to call for active dental interference; but the large majority of these cases are so closely associated with the other structures that they cannot possibly be the cause of serious systemic diseases and remain long unrecognized. A single rooted abscessed tooth may be the unknown cause of chronic headache, and an impacted wisdom tooth may be a common source of earache, and at the same time there may be no local symptoms of pain.

The subject is too large and complicated, however, to make a popular statement that would be comprehensible. All we can say is that American dentistry is doing creditable work in the relief of suffering, and deserves a kinder sympathy and criticism. Surely one ought to be assured of one's facts before making such sweeping criticisms. Dr. Hunter to prove that the highest priced dentists were guilty as well as the lower priced men, cites the case of a rich young man who had a serious case of pernicious anemia from extensive crown and bridge work for which he paid much money. Because a high price was paid it does not necessarily follow that the work was done by a good man. It is well known that rich young men almost invariably have poor teeth, and they do not always patronize skillful dentists, but go to some quack and pay a big fee for what they are lead to believe is a most extraordinary piece of work.

We do not, of course, know how Dr. Hunter has arrived at his conclusions. He may be absolutely fair in his conclusions, but he would have to consider a large number of cases to establish definitely the wild statements he has made. Of the thousands of gold capped teeth comparatively few can be rightfully charged with producing bad systemic results, and many a tooth that would be extracted in England is saved and made useful for years by an American cap crown. Full gold capped crowns are never placed on the visible teeth by careful American dentists, but they are useful for the grinding teeth which are seldom seen, and when the teeth are properly treated they may be crowned without danger. The fault is not with the system, but with those who attempt to use it without having mastered its principles and technique. The American people are better served dentally than any other nation, as our laws require dental practitioners shall be properly educated.

